# Impact of a mindfulness-based intervention on well-being and mental health of elementary school children: results from a randomized cluster trial

**DOI:** 10.1038/s41598-024-66915-z

**Published:** 2024-07-10

**Authors:** Catherine Malboeuf-Hurtubise, Geneviève Taylor, Danika Lambert, Pier-Olivier Paradis, Terra Léger-Goodes, Geneviève A. Mageau, Gabrielle Labbé, Jonathan Smith, Mireille Joussemet

**Affiliations:** 1 Psychology Department, Bishop’s University, Sherbrooke, Canada; 2 Psychology Department, CHUS Research Center, Sherbrooke, Canada; 3 Department of Education and Pedagogy, Université du Québec À Montréal, Montreal, Canada; 4 Psychology Department, Université de Sherbrooke, Sherbrooke, Canada; 5 Psychology Department, Université du Québec À Montréal, Montreal, Canada; 6 Psychology Department, Université de Montréal, Montreal, Canada; 7 Department of Social Work, Université de Sherbrooke, Sherbrooke, Canada; 8 Department of Preschool and Primary Education, Université de Sherbrooke, Sherbrooke, Canada

**Keywords:** Mindfulness-based intervention, Well-being, Basic psychological needs, Mental health, Elementary school children, Program evaluation, Psychology, Human behaviour

## Abstract

Prevention programs, such as mindfulness-based interventions (MBIs), are often implemented in schools to prevent psychological disorders from emerging in children and to support their mental health. This study used a randomized cluster design to evaluate the impact of a MBI, called *Mission Méditation*, on the well-being and the mental health of elementary school children’s. 13 classrooms of an elementary school were randomly allocated to the experimental condition (7 classrooms, *n* = 127 students) or the waitlist control condition (6 classrooms, *n* = 104 students). Participants in the experimental condition received a 10-week MBI. Regression analyses revealed significant differences between conditions for inattention. Participants in the MBI condition reported no change in pre- to post-intervention, whereas participants in the control condition reported pre- to post-intervention increases. Results also showed significant differences in perceived competence. Participants in the MBI condition reported a non-significant decrease in perceive competence, whereas participants in the control condition reported significantly higher perceive competence scores from pre- to post-intervention. Results do not indicate that the MBI had a significant impact on participant’s well-being and mental health. This suggests that MBIs may not have an added value when compared to other preventive interventions geared towards well-being and mental health promotion in school settings.

## Introduction

Stressors derived from the day-to-day life of elementary school-aged children can affect their psychological health^1^. In Canada, approximately 20% of children (aged 5 to 12) suffer from a mental health disorder or significant psychological distress^2^. The most prevalent mental health disorder diagnoses in children are anxiety disorders, which are chronic and persistent beyond childhood^3^. Even more concerning, only one in five Canadian children receive the mental health services they need^4^. This has given rise to researchers and leading experts in education and mental health to propose a focus on well-being promotion and prevention programs for children to be implemented in schools as their delivery is not too onerous and they can prevent such disorders from emerging^4,5^. One intervention avenue that has been explored in the past years are mindfulness-based interventions (MBIs), which have shown promise to improve the mental health of children in elementary school settings. The present study aimed to estimate the impact of a MBI on various mental health indicators of children ages 8 to 12 years old, in a school setting, using a randomized cluster trial (RCT).

### Mindfulness

Although mindfulness is an ancient practice, contemporary research continues to shed light on the various effects it can have in one’s life, namely on mental health and overall well-being^6^. Mindfulness can be defined as “the awareness that emerges through paying attention on purpose, in the present moment, and nonjudgmentally to the unfolding of the experience moment by moment”^7^. Mindfulness brings one’s attention to the present moment in full acceptance of sensations, thoughts, and emotions. This practice has shown to facilitate self-regulation and emotional regulation in adults and youth alike^8^. This can be partly explained by mindfulness’ emphasis on nonjudgmental awareness, as it promotes an engagement with emotions and thoughts that withstands avoidance and rumination. Moreover, mindfulness assumes that mental processes are fleeting phenomena and do not need to be acted upon.

Increasingly, researchers have explored the benefits of different MBIs on mental health. Borkovec^9^ describes the impact of MBIs as an intervention to help individuals learn how to circumvent typical reactions to their emotions—for instance, “holding on to pleasant emotions” and trying to “get rid of or suppress the unpleasant emotions that real or unreal events elicit.” This author argues that these ‘typical’ reactions can distract us from the present moment and can limit genuine emotional processing, as well as distort the information generated by primary affect in the present moment^9^. Indeed, MBIs attempt to teach participants to focus on their present-moment experience in a non-judgmental way, which can help attenuate worrying about past and future events, generally increasing one’s quality of life^10^. Broadly speaking, results from large-scale meta-analyses on studies using RCT designs indicate that MBIs can have small to moderate positive impacts on mental health by reducing anxiety and depressive symptoms in both healthy and clinical adult populations^11–13^.

In recent years, there has been a rapid proliferation of studies examining the impact of MBIs on youth (i.e. children and adolescent) mental health, which promoted the development and implementation of new types of interventions^14^. A mounting body of evidence has demonstrated positive outcomes of MBIs in younger populations, namely with regards to improvements of inattention and hyperactive-impulsive symptoms^15^, externalizing problems^16^, self-regulation difficulties^17^, and rumination^18^. Overall, MBIs designed for youth appear to have positive effects on emotional, behavioral, social, and even physical outcomes^19^, with similar effect sizes as those that have been reported for adults^20^. In children, past research has indicated that MBIs could have a positive impact on emotion regulation, impulsivity, and conduct problems^21^. As such, MBIs can teach children how to identify, process, and express their thoughts and emotions optimally, which can lead to more prosocial behaviour. The positive impact of such prosocial behaviour, in turn, has been linked to better school success and overall well-being^22^.

### MBIs within school settings

When implementing MBIs in school settings, research highlights several positive outcomes when compared to waitlist and active control groups. These include a reduction in internalized problems^23^, depressive symptoms^24^, rumination^24^ and aggressiveness^25^. Moreover, there are reported increases in attention^26^, self-regulation^27^, and even social regulation^25^. Notably, previous studies have associated the decrease in perceived stress, following exposure to an MBI, with improved emotional regulation^25,28^, observed across both boys and girls^29^.

With regards to age, a meta-analysis by Carsley and colleagues^30^ examining school-based mindfulness interventions revealed that interventions aimed at adolescents (i.e., 15 to 18 years old) exhibit the most substantial effects on mental health and well-being, both immediately after the intervention and during follow-up assessments. In contrast, interventions targeted at younger children (i.e., 6 to 10 years old) seemed to demonstrate positive impacts solely at post-test^30^. This trend, wherein older adolescents appear to derive greater benefits from MBIs compared to children, has been echoed in various other studies and reviews^20,31,32^.

Although RCTs have been conducted in this field, much of the available literature on the impact of MBIs on youth mental health relies on studies with small sample sizes and quasi-experimental designs. Moreover, there is no consistent approach across studies to the implementation and training requirements of MBIs in school settings, resulting in the existence of various forms of these interventions. For instance, some interventions vary in length ranging from a single forty-five-minute session^33^, to an intervention comprised of 8 to 10 sessions^34^, and sometimes up to 25 sessions^35^. Although there is no consensus as to the optimal duration of a school-based MBI, the interventions tend to last from 4.5 to 36 h and are ideally made readily accessible to all students in the classroom^36^. The MBIs are sometimes led by an external trained professional or directly by the teacher, who was trained in administering the MBI being tested. Results from Carlsey and colleagues’ meta-analysis^30^ suggests that MBIs delivered by teachers (vs trained professionals) had greater post-test benefits on mental health and wellbeing. Only interventions delivered by teachers had sustained and significant effects in the long term. Further analyses on the types of MBI programs that were most effective revealed that those that included a combination of yoga and mindfulness practices had a greater impact on youth mental health and wellbeing than those reliyng heavily on formal mindfulness practices. Authors hypothesize that existing MBIs may rely too heavily on the extensive training of facilitators, which may not always be feasible or possible in school settings. This, in turn, could have an impact on program adherence, likely reducing potential impacts on youth mental health.

Limitations of the available research thus prevent us from determining, without doubts, whether MBIs really represent an intervention of choice to foster better mental health in a general population of children within school settings. Indeed, a notable disparity exists in the literature regarding the precise nature of this impact. For example, research focusing on clinical populations posits that MBIs may confer greater benefits to children with mental health disorders, given their heightened psychological distress^37^. Conversely, Fung and colleagues^28^ contend that larger MBI treatment gains are primarily observable in children with a lower severity baseline. Studies present conflicting perspectives, making it challenging to conclusively ascertain the influence of age and mental health status on the outcomes of MBIs for youth.

Amidst this discourse, the MYRIAD project^32^ emerges as a pivotal study, representing the most extensive RCT of MBIs conducted among early adolescents (mean age = 12 years-old) in classrooms in the UK. However, it also raises concerns regarding potential harm. Indeed, MYRIAD researchers suggested that MBIs may be contraindicated for early adolescents exhibiting symptoms of poor mental health or those at risk of developing such symptoms. Additionally, their findings highlight challenges faced by younger adolescents (aged 11 and younger) in acquiring and applying mindfulness skills compared to slightly older teenagers due to underdeveloped metacognitive abilities.

Nonetheless, it has been suggested that future MBI research should focus on tailoring programs specifically to elementary school children, namely, to increase student acceptability and receptivity and to develop beneficial prevention interventions to foster greater well-being and prevent mental health problems in this population. RCTs are thus needed to better isolate and assess the impact of MBIs as RCTs are considered the gold standard to examine the efficacy of interventions.

### Mindfulness and self-determination theory (SDT)

Few theoretical models exist to explain why and how MBIs have a positive impact on youth’s well-being and mental health^38^. On one hand, theories and models have been proposed to explain the self-regulatory and neuropsychological effects and mechanisms of mindfulness among adults^39–41^. On the other hand, the few conceptualizations that have been developed for children are mostly based on practical, intervention development frameworks for MBIs^42,43^, but do not provide grounded theoretical, testable predictions regarding mindfulness and its expected effects.

Self-determination theory (SDT)^44–46^, is applicable to individuals of all ages and can guide predictions about the impact of mindfulness on children’s well-being. SDT is a macro-theory of human functioning, which posits that the health, functioning, and well-being of all people, adults and children alike, is promoted when their innate, basic psychological needs (BPN) for competence (a sense of mastery and efficacy), relatedness (a sense of interconnectedness to others), and autonomy (a sense endorsement of one’s behaviors) are met. Decades of research have shown that social contexts that fulfill these three BPN support better mental health, both in children with and without learning and mental health challenges^47^. Overall, the satisfaction of these three basic needs is considered essential for the promotion of personal growth and well-being^45^.

MBIs have been brought forward as promising interventions to help people increase their own BPN satisfaction. Indeed, some SDT researchers consider MBIs to have the potential to increase one’s awareness of mental phenomena (e.g., urges, emotions and desires) and external conditions (e.g., pressures and conflicts), which in turn could foster the level of reflection and volition that is conducive to greater BPN satisfaction^48–50^.

The available research linking mindfulness and BPN shown that trait and state mindfulness seem to favor better BPN satisfaction and well-being^48,49,51^. This research, which is generally correlational, has been done almost exclusively among adult samples. Other studies on mindfulness and SDT have rather focused on the concept of autonomous motivation, which is related to, but not the same as, BPN^52^. To our knowledge, only a few studies^53,54^ have examined the effects of mindfulness training on students’ BPN. The few published intervention studies that have been conducted in school settings with children were done with students with severe learning disabilities (LDs) and have shown inconsistent results. Indeed, in a quasi-experimental design, Malboeuf-Hurtubise and colleagues^21^ noted that the *Mission Méditation* program decreased overall BPN among a sample of 14 students with severe LDs, aged between 9 and 12 years old. The same authors conducted a RCT with an active control condition in a similar sample and found that students assigned to this same program showed an increase in competence at the within-subject level at post-test, but a similar increase was also found among children in the active control condition^55^.

While these studies provide valuable insights, they have notable limitations that must be acknowledged. First, only one of the studies employed a controlled design, limiting the ability to establish causal relations. Second, both studies focused on small samples of children with severe LDs, preventing the generalization of the findings to a broader school-aged child population. To gain a better understanding of the impact of MBIs, further research using RCT designs with larger and more diverse samples of students is needed. Such studies may allow to better capture the effects of MBIs on child mental health in a community sample.

### Present study

The present study responds to this need and analyzes the short-term effects of a MBI called *Mission Méditation* on elementary school children’s well-being and mental health indicators. As such, a waitlist RCT design was used, in which a MBI was offered to elementary school students from 3rd grade to 6th grade who were randomly assigned to the experimental or the waitlist control conditions.

### Primary hypotheses

We hypothesized that the delivered MBI would have a pre-to-post intervention effect on BPN satisfaction and on various indicators of mental health. Specifically, we expected that the children randomly assigned to the experimental MBI condition would show reduced anxiety, depression, and inattention, as well as improvements in autonomy, relatedness, and competence compared to the children in the waitlist control condition. Students assigned to the control condition were not expected to have significant changes in scores from pre-to-post intervention time points.

## Methods

### Design

The present study obtained ethics approval from Université du Québec à Montréal’s Research Ethics Board (file # 2026_e_2017). All methods were performed in accordance with Declaration of Helsinki. We used a RCT with a waitlist control condition in one elementary school in the suburbs of a large city in the province of Quebec, Canada. The RCT was registered on 21/03/2024 on the ClinicalTrials.gov platform (Identifier: NCT06346002). A total of 13 classrooms were randomly allocated (1:1 ratio) to either the experimental condition (7 classrooms, *n* = 127 students) or the waitlist control condition (6 classrooms, *n* = 104 students), using a random numbers table. The project senior research assistant oversaw the randomization and assignment of groups to each condition. Both groups filled out pre- and post-intervention measures at the same time points, in class, to minimize attrition. Participants assigned to the experimental condition received a 10-week MBI from September to December. Children in classrooms randomly assigned to the waitlist condition received the same intervention from January to March, once all pre- and post-intervention measures were completed.

### Participants

A total of 13 classrooms of 3^rd^ to 6^th^ grade elementary school students (*N* = 231; aged 8 to 12 years old [*M*_age_ = 9, 87 years old]; 51% boys and 49% girls), were initially recruited to take part in this study. Seven classrooms were initially randomly allocated to the experimental condition, while six classrooms were allocated to the control condition. Parents and teachers of participating children provided informed consent for their children to participate in the current study. However, despite attempts to engage parents and secure their consent, their lack of participation in completing questionnaires resulted in their data being ineligible for analysis. We did not have additional demographic information for study participants, outside of sex and age.

Randomization occurred after completion of pre-intervention measures. Two assessment time points were scheduled: pre-intervention (one week prior to the beginning of MBI delivery to experimental classrooms) and post-intervention (immediately after the end of the MBI delivery). Two research assistants helped students, during class time, to fill out their questionnaire, reading all items out loud and answering questions as needed.

### Intervention and implementation

The implemented MBI program, called *Mission Méditation,* comprises ten weekly sessions^56^. This MBI is specifically tailored for elementary school children. Activities comprised in this intervention encompassed formal (e.g., body scan, sitting, and breathing meditation) and informal meditations (e.g., mindful eating, listening, and touching, mindful walking), as well as positive psychology exercises (e.g., taking care of oneself, gratitude). Further details about this program can be found here^34^. The weekly sessions, lasting 45 to 60 min each, were delivered in a group format by the teachers themselves, in their classroom. Teachers in the experimental condition were asked to practice concepts with their students at least once a week between sessions. Teachers were all previously trained, over the course of a three-day period (for a total of 24 h of training), by the first and second authors (CMH and GT). Teachers were also asked to fill out a weekly journal indicating which activities were completed and whether practice occurred in between sessions, to monitor intervention implementation.

### Measures

#### Symptoms of mental health disorders

Children completed selected items from the self-report version of the anxiety (3 items, e.g., “I worry about little things”), depression (5 items, e.g., “Nothing ever goes right for me”) and inattention (4 items, e.g., “I forget to do things”) subscales of the *Behavior Assessment Scale for Children* (BASC II^57^;). The BASC-II is a comprehensive mental health and behavioural scale which is extensively used and validated in clinical settings with youth. Items from selected subscales were selected—as opposed to using the full subscales—to ensure that participants could fill out the questionnaire package in a timely and developmentally appropriate fashion. Internal consistency was acceptable^58,59^ for all subscales at pre-intervention and post-intervention (α_depression_ = 0.81/0.85; α_anxiety_ = 0.70/0.77; α_inattention_ = 0.76/0.77).

#### Basic psychological needs satisfaction scale

Participants rated how competent, autonomous, and related they felt in school, by answering a 9-item scale adapted from a scale used in a previous, similar study^58–61^. Adaptations included changes in wording to be easily understandable by children. Children were asked to rate their agreement with items such as “In school, I feel free to be myself” (autonomy); “I am able to reach my goals” (competence) and “In my relationship with others, I feel appreciated” (relatedness) on a 5-point Likert scale ranging from 1 (almost never) to 5 (almost always). Internal consistency at pre-intervention and post-intervention was acceptable in this sample for competence (α_competence_ = 0.62/0.64) and relatedness (α_relatedness_ = 0.66/0.75). However, the reliability of the autonomy construct was unacceptable (α_autonomy pre/post_ = 0.12/0.29), and, as such, this variable was discarded from further statistical analyses.

#### Process measure

Finally, participants completed the Mindful Attention and Awareness Scale for Children (15 items; e.g., “I find it hard to stay focused on what’s happening in the present moment.”; Ref.^60,61^) to evaluate pre-to-post changes in their mindfulness abilities. They rated their agreement with each item on a 6-point Likert scale ranging from 1 (never) to 6 (almost always). A higher score on this scale indicates lower levels of mindfulness. Internal consistency was acceptable in this sample (α_pre/post_ = 0.69/0.67).

### Data analysis

Descriptive statistics are presented separately for each condition considering pre-to-post-intervention scores. Categorical data are presented with frequencies and percentages. Continuous data with normal distribution are presented as means with standard deviation (*SD*), while non-normal data are presented as medians with interquartile ranges. Quantile-quantile plots and histograms were used to assess normality. All variables were normal. We considered a significance level of 5%. Please refer to Table 1 for means and standard deviations of pre-to-post scores across groups.

**Table 1:** Means and standard deviations for pre-to-post intervention scores.

Measure	Intervention (*n* = 127)		Control (*n* = 104)	
	Pre	Post	Pre	Post
Anxiety	3.23 (1.87)	3.6 (2.06)	3.2 (2.05)	3.43 (2.1)
Depression	3 [1–5.75]	4 [1–5.25]	3 [1–5]	3 [1–5]
Inattention	3 [2–5]	3 [1.5–4]	3 [1–5]	3.5 [1–5]
Competence	3.33 [2.33–5.33]	3 [2.33–5.33]	3 [2.33–5.33]	4.42 [2.67–5.58]
Relatedness	3.08 (1.05)	3.21 (1.13)	3.26 (0.96)	3.22 (0.94)
Mindfulness	2.33 [1.83–3]	2.5 [2–3.3]	2.33 [1.83–3]	2.42 [2–3.13]

Descriptives presented as mean (SD) or median [IQR].

To assess within-person differences within conditions for each outcome, linear regression models were used. We considered post-test scores as dependent variables explained by condition and pre-test scores as the independent variables. This method allowed us to adequately compare post-test outcomes between conditions while adjusting for baseline differences (and the regression-toward-the-mean artefact). Results are presented as unstandardized (mean differences) and standardized (strength of association) coefficients and their 95% confidence interval.

As sensitivity analyses, paired t-tests were conducted within condition to document pre-to-post differences. Assumptions of normally distributed residuals and homoscedasticity were verified visually with appropriate diagnostic plots; all variables were normal. Statistical analyses were conducted using SPSS v.26 (IBM Corporation, Armonk, NY, USA) and R v.4.0.2 (R Core Team, Vienna, Austria).

## Results

A total of 231 students were included and no participants were excluded. A total of 127 children received the intervention and 104 were controls. Results of the regression analyses for each main outcome are presented in Table 2. Linear regression models revealed a significant difference between conditions for inattention (β = −0.67 [−1.25, −0.08], *p* = 0.03) and competence (β = −0.52 [−0.82, −0.22], *p* = 0.001). There was no statistically significant difference between conditions on any other dependent variable. While participants in the MBI experimental condition did not report significant changes in inattention scores from pre-intervention to post-intervention, participants from the waitlist control condition reported higher inattention scores from pre-intervention to post-intervention.

**Table 2:** Results from regression linear models for main outcomes.

Measure	Coef	C.I. 95%		P-value
Anxiety				
Between-group differences	0.16	−0.32	0.64	0.53
Pre-test	0.55	0.43	0.68	< .001
Depression				
Between-group differences	0.45	−0.36	1.26	0.28
Pre-test	0.60	0.48	0.72	< .001
Inattention				
Between-group differences	−0.67	−1.25	−0.08	0.03
Pre-test	0.57	0.45	0.68	< .001
Relatedness				
Between-group differences	−0.11	−0.33	0,1	0.30
Pre-test	0.54	0.44	0.64	< .001
Competence				
Between-group differences	−0.52	−0.82	−0.22	0.001
Pre-test	0.71	0.62	0.81	< .001
Mindfulness				
Between-group differences	0.12	−0.09	0.33	0.26
Pre-test	0.63	0.51	0.74	< .001

Sensitivity analyses then showed that while participants in the MBI experimental condition did not report significant changes in inattention scores from pre-intervention to post-intervention (*t*(107) = 0.84, *p* = 0.39 ), participants from the waitlist control condition reported significantly higher inattention scores from pre-intervention to post-intervention (*t*(93) = −2.25, *p* = 0.02). As for competence, descriptive statistics showed that while participants in the MBI experimental condition reported a non-significant decrease in their competence scores from pre-intervention to post-intervention (*t*(117) = 0.05, *p* = 0.96), participants from the waitlist control condition reported higher competence scores from pre-intervention to post-intervention (*t*(99) = −3.6, *p* =  < 0.001). We found no significant pre-to-post differences within conditions for anxiety, depression, or mindfulness.

## Discussion

The goal of this study was to evaluate the impact of a MBI on well-being and mental health of 8- to 12-year-olds, using a waitlist RCT design. Our findings did not directly support our hypotheses; results from regression analyses suggest that this specific MBI did not have a significant impact on mindfulness and child mental health, and there is even a possibility of negative impact on their BPN for competence. These results echo findings from a previous study conducted by our research team, in which a MBI had a detrimental impact on BPN satisfaction in elementary school children with severe learning disabilities^58,59^. Furthermore, our findings align with the extensive research conducted in the MYRIAD project^32^ which involved a nationwide randomized controlled trial of an MBI among early adolescents in the UK. Their study revealed adverse effects of the evaluated MBI on some students, leading them to conclude that MBIs may not be suitable as a universal intervention in a school setting. In fact, they even suggested that MBIs might be contraindicated for early adolescents exhibiting symptoms of poor mental health or those in the process of developing such symptoms.

Among the measures examined in this study, only inattention and competence yielded statistically significant results. Inattention scores from the MBI condition showed no significant change from pre-to-post intervention, while scores from the waitlist control condition increased. This could indicate that students tend to get more inattentive as the academic year progresses^62,63^, and that the MBI may have had a protective effect in that it stabilized their attention levels. In other words, it avoided a worsening of their attention over time. However, the MBI did not lead to improvements in inattention scores in the experimental condition, contrary to findings reported in other mindfulness studies where teachers observed significant attention improvements following mindfulness interventions^15^.

Moreover, concerning competence, participants’ scores from the MBI condition decreased – indicating a worsening of their satisfaction of the need for competence—whereas scores from their waitlist counterparts increased. However, sensitivity analyses showed this decline was not statistically significant. This unexpected finding raises questions about the underlying mechanisms at play. One plausible explanation is that the practice of mindfulness might remove barriers that previously hindered accurate self-assessment of BPN satisfaction, including the need for competence^64,65^. Consequently, children may become more aware of their own limitations, particularly in terms of regulating their difficult emotions, potentially leading them to experience a heightened sense of incompetence within the school context. This increased awareness might also lead them to notice the emphasis placed on evaluation in school, something that has often been criticized by education experts in SDT^62,63^. A general school context that is focused on evaluation tends to be controlling, which leads to a thwarting of autonomy, competence, and relatedness needs in children^62,63^. It is possible that by making children more aware of their surroundings, and in turn, of the way in which adults in the school address them and act with them, the MBI led to a more realistic perception of their feelings of competence at school, which was lower than they had previously thought. Furthermore, another potential explanation for the decrease in competence feelings in the MBI condition is that the teachers who were part of the waitlist control condition might have unconsciously adjusted their teaching to compensate for the fact that they were not doing mindfulness training with their students. On the other hand, the teachers who *were* giving the MBI to their students might have paid less attention to the way in which they provided competence support in their class *because* they were already using an intervention. Further research is needed to substantiate and refine these hypotheses.

To further explore these findings, it is important to consider additional factors that could contribute to the observed decline in competence scores. Although we did not have access to information pertaining to the presence of diagnosed psychological disorders or learning disabilities in this sample (all students were in regular classrooms), it could be that some youth for whom competence scores decreased from pre-to-post intervention presented more difficulties in school than their counterparts^18^. Further studies may want to document the presence of such diagnoses to further our understanding of how MBIs impact children with different psychological profiles and determine if this type of intervention should not be recommended for specific subgroups of students.

Building upon this insight, it is important to consider the developmental stage of elementary school students when interpreting these findings. As noted by Jennings^31^, an international leading figure on mindfulness research in the school setting, MBIs may be developmentally inappropriate for elementary school students, hindering their ability to fully benefit from mindfulness teachings. This is because certain required abilities, such as substantial metacognitive ability, only develop later in their lives. Indeed, the MYRIAD project^32^ supports this perspective, suggesting from their data that younger adolescents (aged 11) may face challenges in acquiring and applying mindfulness skills compared to slightly older teenagers due to underdeveloped metacognitive abilities. Importantly, the authors also point out potential challenges in self-regulating behavior for younger adolescents, particularly in managing challenging thoughts and emotions evoked during mindfulness practices.

Extrapolating from these findings, it is reasonable to infer that children younger than 11 may face similar limitations. Consequently, when confronted with a sense of incompetence arising from a mindfulness exercise in class, a child may struggle to cope effectively with the realization of their limitations, lacking the means for appropriate actions^32^. In contrast, adults are more likely to possess the agency and skills to address their perceived limitations. This suggests a potential detrimental impact of MBIs on children's competence levels, which might explain the observed decline in competence scores. It raises questions about the suitability of MBIs as interventions to enhance BPN satisfaction in elementary school students.

The observed “worsening phenomenon”, characterized by a decline in indicators of mental health following participation in an MBI, has been observed in previous studies. These studies have documented various negative effects, including temporary increases in anxiety, higher self-reported hyperactivity/inattention, elevated scores in panic disorder and obsessive-compulsive symptoms, as well as detrimental effects on overall well-being and an increased risk of depression^32,64–66^. It is noteworthy that some of these effects have been observed not only immediately after the intervention but also during the 1-year follow-up period.

To counteract these potential negative effects of MBIs in the school setting, one solution would be to implement other approaches to foster better coping strategies in children^55,67^. For instance, social-emotional learning (SEL) programs could permit children to learn to regulate the difficult emotions they were taught to identify^68–71^. This has been suggested by researchers^68–71^, and studies focusing on evaluating these are currently in progress (e.g., the Compassionate Schools Project described in^68,70^). By teaching social-emotional skill-building activities, children may develop the necessary agency and resilience to effectively cope with the awareness of their thoughts and feelings, mitigating potential negative effects.

A factor that may contribute to the overall lack of efficacy of the MBI in improving mental health outcomes among children relates to its delivery. Despite providing teachers with 24 h of training, encompassing personal mindfulness practice and instructions on delivering the MBI to students, and considering feedback from teachers with regards to treatment adherence, it remains challenging to determine the extent to which each teacher demonstrated the competencies outlined in the MBI Teaching Assessment Criteria (MBI:TAC; Ref.^72^) during the implementation of our MBI. These criteria encompass not only adherence to the mindfulness program's curriculum but also relational skills, the embodiment of mindfulness by the teacher, the quality of guidance, and the effectiveness of interactive inquiry. Although all teachers received training in these skills, it is plausible that some teachers developed them to a lesser extent than others, potentially resulting in decreased competence in leading MBI sessions. For instance, during training, it was acknowledged that it can be arduous for teachers to shift away from the mindset of seeking the “correct response.” When teaching mindfulness and engaging in discussions about children’s experiences, most teachers need to consciously inhibit the automatic inclination to search for the “right way” to feel after a meditation exercise^73,74^. It is conceivable that additional hours of training in personal mindfulness practice could be necessary to assist teachers in cultivating greater acceptance and non-judgment toward their own experiences, thereby enhancing their ability to effectively teach mindfulness to children.

Indeed, several studies evaluating the Cultivating Awareness and Resilience in Education program (CARE; Ref.^73,74^) have demonstrated the positive effects of mindfulness-based training for teachers. These studies reveal that teachers who participate in such training experience a range of benefits, including reduced psychological distress, fewer physical symptoms, and improved emotion regulation. In turn, these teachers exhibit increased unconditional emotional support and better organizational support for their students^75,76^. Importantly, the program also appears to have a positive effect on the teachers’ students, as they become more engaged in class, more motivated, and improve their reading skills^77,78^. This suggests a notable “trickle-down” effect, where students reap the benefits of teachers whose personal mindfulness skills enhance their emotional responsiveness towards their students, despite never having been taught mindfulness techniques themselves. What is also important to note here is that unconditional emotional support represents one of the need-supporting behaviours that has been shown to increase satisfaction of the BPN for autonomy, competence and relatedness in SDT education research^62,63,79^.

Our results, coupled with the MYRIAD study and Jennings’^31^ commentary, suggest that the training of teachers in mindfulness-based practices should be prioritized rather than focusing on the widespread implementation of MBIs in school settings. Findings suggest that mindfulness practice enables teachers to enhance their own well-being, leading to indirect improvements in the learning outcomes and well-being of their students. This approach offers a potential solution to circumvent the emerging adverse effects observed in studies on mindfulness-based school programs that predominantly concentrate on teaching mindfulness skills to children.

## Strengths and limitations

A significant strength of this study is its strong methodology. By recruiting over 200 elementary school children of varying ages, the study ensured that the obtained results could support robust analyses. This is particularly noteworthy considering that many previous evaluations of MBIs offered to children relied on smaller sample sizes, whereas we can confidently say that this study had sufficient statistical power. Additionally, the study employed a RCT design, which is a strong methodology for evaluating intervention efficacy. This design minimized bias and increased internal validity by randomly assigning classrooms to either the experimental or control conditions.

Moreover, this study builds upon existing literature by addressing gaps in research on MBIs with elementary school-aged children. Few RCTs have focused on MBIs in this population, and even fewer studies have examined the effects of mindfulness interventions on BPN. This study is notable as it is among the largest-scale investigation of an MBI's impact on BPN satisfaction, specifically among children. The study also offers a new perspective by reporting negative impacts on mental health following participation in an MBI. This contrasts with previous scientific articles that have generally highlighted the benefits of MBIs, and it aligns with recent publications cautioning against widespread implementation of MBIs with children in the school setting^31,32^.

Lastly, a notable strength of our study is the unique design of the MBI used, which was specifically developed for children rather than being adapted from interventions designed for adults. Jennings^31^ sheds light on the prevalent practice of employing mindfulness-based practices meant for adults with children and adolescents, assuming comparable benefits. However, she emphasizes the necessity of considering developmental prerequisites, such as meta-cognition and specific attentional capacities, for children to fully engage in mindfulness practices and experience their associated benefits. In contrast, our study implemented a MBI explicitly tailored to the needs of children, fostering its potential to improve the mental health of young students. Despite our study yielding unexpected and divergent results from prior research findings, the deliberate utilization of a MBI designed for children significantly bolsters the internal validity of our conclusions.

Several limitations should be acknowledged in this study. First, the lack of multi-informant data hampers a comprehensive understanding of the intervention’s effects. Despite efforts to involve parents and obtain their consent, their disengagement in filling out questionnaires rendered their data unanalyzable. Including parent-reported information would have provided valuable insights and nuanced the interpretation by allowing comparisons with self-reported scores from children. Prior mindfulness studies have demonstrated the importance of multiple perspectives, as outcomes can vary when children’s self-reported measures are contrasted with those reported by teachers or parents^15^. The length of the parent questionnaire, taking approximately 20 min to complete, might have hindered their full engagement. Future research should explore strategies to enhance and retain parental participation, such as shortening the questionnaire or offering participation incentives within budget constraints.

Another limitation concerns the utilization of subscales rather than complete scales within this study. For example, we were unable to analyze the impact of the MBI on the satisfaction of the need for autonomy. The low reliability coefficients of certain other scales imply that some questions may not have been fully understood by the children, potentially introducing bias into the results. To enhance the validity and reliability of measures, future studies should consider using scales with additional subscale items, instead of relying solely on subsets of each scale.

Moreover, to complement self-report questionnaires, future studies could use projective measures with children who may struggle with impression management or lack the necessary reading, writing, and introspection skills to fill out questionnaires. In fact, some children and even adults may lack the self-access necessary to reliably evaluate their own situation^80^. Notably, Katz and colleagues^81^ have validated an adapted version of the Thematic Apperception Test (TAT) for measuring autonomous motivation in fifth-grade children. Their research demonstrated that the projective measure was more sensitive to experimentally induced changes in autonomous motivation compared to the corresponding self-reported questionnaire. This suggests that assessing autonomy satisfaction through pictures that children describe may provide a more accurate measure than directly asking them about their autonomy satisfaction. Furthermore, ongoing research utilizing the research version of the TAT, the Picture-Story Exercise (PSE; Ref.^82^), is currently underway to measure the need for autonomy in both adults and children^83^. This line of research holds promise for future studies seeking to assess the impact of MBIs on autonomy and other psychological need satisfaction. Other tools, such as the Robert’s Apperception Test, may also be of use for such a purpose.

Furthermore, the absence of an active control condition represents a limitation in this study. While the inclusion of a waitlist control condition enabled controlling for time elapsing, the lack of an active control condition makes it challenging to attribute the obtained effects specifically to the evaluated MBI. This limitation prevents us from definitively identifying which aspects of the intervention were beneficial and which may have contributed to poorer outcomes.

Additionally, it is important to note that this study did not include a follow-up assessment after the post-intervention measurement. Therefore, the long-term effects (or lack thereof) of the MBI on indicators of mental health in school-aged children remains unknown. Further research should explore whether the null and negative effects observed in this study persist over time and investigate the potential long-term impacts of MBIs on children's need satisfaction, mindfulness and mental health outcomes.

Lastly, the generalizability of the findings is limited due to the study's focus on a single elementary school within a specific province in Canada. Factors such as cultural differences and regional variations can influence the outcomes of MBIs in diverse contexts. For instance, a recent systemic review and meta-analysis by Sun and colleagues^84^ found that people of color tend to derive comparatively lesser benefits from MBIs, as evidenced by lower effect sizes. This pattern persists across different populations, including psychiatric patients, medical subjects, and both healthy adults and youth^84^. In addition, meta-analyses have revealed variability not only across geographical locations within the United States^85^, but also across multiple countries^86^. To enhance external validity, future research should aim to include samples from diverse geographical locations, allowing for a broader understanding of the applicability of the findings across different settings and populations.

## Conclusion

Results from this longitudinal study did not show that the studied MBI, delivered to 3rd to 6th graders by their teachers, had significant positive effects on BPN and various indicators of mental health. On the contrary, the MBI seemed to have a detrimental effect on the satisfaction of the basic need for competence at post-test. As such, results from this larger scale study do not support the effectiveness of MBIs to foster better mental health in elementary school students. However, a lack of multi-informant data and the use of selected scale items in this project are limitations of this study. The lack of an active control group, follow-up data and the fact that this project was implemented in one single school also impacts the generalizability of these results. Future research should consider evaluating the long-term effects of MBIs in children, whether MBIs should be implemented with teachers vs. students, as well as the added value of MBIs in comparison to other forms of interventions (such as SEL-based programs) that can be delivered in school settings, with an easier implementation process and at a lower cost.

## Data Availability

Datasets will be available upon request. For further information, please contact Catherine Malboeuf-Hurtubise: catherine.malboeuf-hurtubise@ubishops.ca.
